# Dorsal and ventral horn atrophy is associated with clinical outcome after spinal cord injury

**DOI:** 10.1212/WNL.0000000000005361

**Published:** 2018-04-24

**Authors:** Eveline Huber, Gergely David, Alan J. Thompson, Nikolaus Weiskopf, Siawoosh Mohammadi, Patrick Freund

**Affiliations:** From the Spinal Cord Injury Center (E.H., G.D., P.F.), Balgrist University Hospital, Zurich, Switzerland; Department of Brain Repair and Rehabilitation (A.J.T., P.F.) and Wellcome Trust Centre for Neuroimaging (N.W., S.M., P.F.), UCL Institute of Neurology, University College London, UK; Department of Neurophysics (N.W., P.F.), Max Planck Institute for Human Cognitive and Brain Sciences, Leipzig, Germany; and Department of Systems Neuroscience (S.M.), University Medical Center Hamburg-Eppendorf, Germany.

## Abstract

**Objective:**

To investigate whether gray matter pathology above the level of injury, alongside white matter changes, also contributes to sensorimotor impairments after spinal cord injury.

**Methods:**

A 3T MRI protocol was acquired in 17 tetraplegic patients and 21 controls. A sagittal T2-weighted sequence was used to characterize lesion severity. At the C2-3 level, a high-resolution T2*-weighted sequence was used to assess cross-sectional areas of gray and white matter, including their subcompartments; a diffusion-weighted sequence was used to compute voxel-based diffusion indices. Regression models determined associations between lesion severity and tissue-specific neurodegeneration and associations between the latter with neurophysiologic and clinical outcome.

**Results:**

Neurodegeneration was evident within the dorsal and ventral horns and white matter above the level of injury. Tract-specific neurodegeneration was associated with prolonged conduction of appropriate electrophysiologic recordings. Dorsal horn atrophy was associated with sensory outcome, while ventral horn atrophy was associated with motor outcome. White matter integrity of dorsal columns and corticospinal tracts was associated with daily-life independence.

**Conclusion:**

Our results suggest that, next to anterograde and retrograde degeneration of white matter tracts, neuronal circuits within the spinal cord far above the level of injury undergo transsynaptic neurodegeneration, resulting in specific gray matter changes. Such improved understanding of tissue-specific cord pathology offers potential biomarkers with more efficient targeting and monitoring of neuroregenerative (i.e., white matter) and neuroprotective (i.e., gray matter) agents.

Spinal cord injury (SCI) usually leads to sensorimotor dysfunction resulting from damage at the level of injury. However, a complex cascade of secondary neurodegenerative processes occur across the spinal cord and brain.^1^ In chronic SCI, cervical cord atrophy of up to 30% has been reported above the level of injury; its magnitude relates to the degree of clinical impairment.^2^ Recent improvements in diffusion-weighted imaging and anatomic sequences with higher in-plane resolution,^3^ combined with advanced postprocessing techniques,^4,5^ now allow the assessment of gray and white matter changes in the cervical spinal cord occurring after SCI.

Although white matter pathology within the spinal cord contributes to sensorimotor impairments, the functional effects of gray matter pathology above the level of injury are uncertain. Improved understanding of tissue-specific cord pathology may allow more efficient targeting and monitoring of neuroregenerative and neuroprotective agents. This study therefore addresses to what extent cord atrophy above the level of injury is driven by pathophysiologic processes occurring in gray and white matter, whether lesion severity is associated with the magnitude of neurodegeneration above the level of injury, and whether the tissue-specific neurodegeneration is associated with neurophysiologic and clinical outcome.

Using structural and diffusion MRI data, we assessed tissue-specific cord pathology above the level of injury in patients with chronic SCI compared to healthy controls. These measures included the assessment of dorsal horn area (DHA) and ventral horn area (VHA),^6^ diffusivity changes within the major spinal pathways, and associations between lesion severity,^7^ tissue-specific pathology, and neurophysiologic changes.

## Methods

### Standard protocol approvals, registrations, and patient consents

Our study protocol was designed in accordance with the Declaration of Helsinki and was approved by the local ethics committee of Zurich (KEK-ZH-Nr. 2012-0343, PB_2016-00623). All participants gave their written informed consent before participation.

### Participants

We recruited 17 patients with SCI (mean age 48.7 ± 14.1 years, 3 female patients) between November 2014 and May 2016 who were preciously admitted to the University Hospital Balgrist (Zurich, Switzerland). Twenty-one healthy controls (mean age 41.7 ± 11.3 years, 7 female controls) from the local neighborhood served as a control dataset that was acquired and used in a previous study.^8^

Inclusion criteria for patients with SCI were traumatic cervical SCI, no other neurologic or mental disorders affecting clinical outcome, age between 18 and 70 years, MRI compatible, and no pregnancy.

### Clinical assessments

All patients were examined with comprehensive clinical protocols to assess neurologic and functional impairment. These included the International Standards for Neurological Classification of Spinal Cord Injury protocol for motor, light-touch, and pinprick score and completeness of injury^9^; the Spinal Cord Independence Measure (SCIM) to measure daily life independence^10^; the Graded Redefined Assessment of Strength, Sensibility and Prehension (GRASSP) for assessing upper limb function^11^; and the Walking Index for Spinal Cord Injury (WISCI).^12^ All patients completed the full protocol, except GRASSP score was not available for 1 patient.

### Neurophysiologic assessments

Contact heat evoked potentials (CHEPs) and somatosensory evoked potentials (SSEPs) were acquired bilaterally in patients at the dermatomes C4, C6, and C8 to measure the integrity of the spinothalamic tract (i.e., CHEPs) and the dorsal column (i.e., SSEPs). For the acquisition of CHEPs^13^ and SSEPs,^14^ the same protocols were applied as previously described.

### Contact heat evoked potentials

A contact heat stimulator (PATHWAY Pain & Sensory Evaluation System, Medoc, Ramat Yishay, Israel) was used to deliver contact heat stimuli from a baseline temperature of 35°C to a peak temperature of 52°C with a heating rate of 70°C/s and a cooling rate of 40°C/s. For each dermatome, we first assessed heat perception and pain thresholds within 2 consecutive trials. For the CHEPs recording, scalp recording sites were prepared with Nuprep (D.O. Weaver & Co, Aurora, CO) and alcohol. Three 9-mm Ag/AgCl surface disk electrodes were positioned according to the international 10-20 system with the active electrode at the Cz position and referenced to linked earlobes (A1–A2); impedances were kept <5 kΩ. Ten to 15 contact heat stimuli were applied (interstimulus interval 8–12 seconds). Two seconds after each stimulus, an audio cue appeared, and patients rated their perceived intensity according to a numeric rating scale. All signals were sampled from 100 milliseconds before the trigger to 1,500 milliseconds after the trigger at a sampling rate of 2,000 Hz with a preamplifier (20,000× bandpass filter = 0.25–300 Hz; ALEA Solutions, Switzerland). Data were recorded in a LabView-based program (V1.43 CHEP; ALEA Solutions, Zurich, Switzerland) with a 100-millisecond period before the trigger and 1-second posttrigger period. Raw data were bandpass filtered from 0.5 to 30 Hz.

### Somatosensory evoked potentials

For dermatomal SSEPs, Key Point (Medtronic, Mississauga, ON, Canada) was used to record and deliver electric stimulation of 3 Hz. Stimuli were elicited by single 0.2-millisecond, repetitive, square-wave electric stimulation. We first assessed electric perception and pain thresholds for each dermatome (not exceeding 40 mA) within 2 consecutive trials. For the recording of SSEPs, surface gel electrodes (10 mm) were used on each dermatome after the skin was prepared with Nuprep (D.O. Weaver & Co) and alcohol. Disposable needle electrodes (Spes Medica, Srl, Genova, Italy) were positioned according to the international 10-20 system with the active electrode positioned at the contralateral side for the stimulated dermatome (C3-4) referenced to Fz; impedances were kept <5 kΩ. The stimulation intensity was individually set as 3-fold electric perception threshold. Averages of 2 traces of 300 cortical responses were obtained for each dermatome. Raw data were bandpass filtered from 2 to 2,000 Hz.

### Neurophysiologic classification

We determined amplitudes and latencies of each dermatome for each patient after averaging all single-trial waveforms for CHEPs (i.e., N2P2, N2, P2) and SSEPs (i.e., N1P1, N1, P1).

Furthermore, CHEPs and SSEPs were classified as normal (onset latency ≤2 SDs from control dermatome recording), pathologic (onset latency >2 SDs from control dermatome recording), or absent (not recordable).^14^ The CHEPs protocol was acquired fully in 14 patients and partially in 1 patient. For SSEPs, 12 patients received the full protocol and 2 patients participated in part of the protocol.

### Image acquisition

All imaging was performed on a clinical 3T Skyra^Fit^ scanner (Siemens Healthcare, Erlangen, Germany) equipped with a 16-channel radiofrequency receive-only head and neck coil and a radiofrequency body transmit coil. A stiff neck (Laerdal Medicals, Stavanger, Norway) was used in all participants to minimize motion artifacts. As a result of motion artifacts, 1 patient was excluded from macrostructural analysis, and 3 patients had to be excluded from microstructural analysis.

At the lesion epicenter, a sagittal T1-weighted (repetition time 600 milliseconds, echo time 9.9 milliseconds, flip angle 150°, in-plane resolution 0.57 × 0.57 mm, slice thickness 3.3 mm), a sagittal T2-weighted (repetition time 3,500 milliseconds, echo time 84 milliseconds, flip angle 160°, in-plane resolution 0.34 × 0.34 mm, slice thickness 2.75 mm), and an axial T2-weighted image (repetition time 5,510 milliseconds, echo time 93 milliseconds, flip angle 150°, in-plane resolution 0.5 × 0.5 mm, slice thickness 3.6 mm) were acquired to assess the lesion size.

At the cervical cord above the level of injury (centered at C2-3), 5 volumes were acquired with a T2*-weighted 3-dimensional multiecho gradient recall echo sequence (multiple echo data image combination^15^) in the oblique axial plane (i.e., perpendicular to the cord) to assess gray and white matter atrophy. Each of the 5 volumes acquired consisted of 20 partitions with a resolution of 0.5 × 0.5 mm^2^ (field of view 192 × 162 mm^2^, slice thickness 2.50 mm [10% gap], repetition time 44 milliseconds, echo time 19 milliseconds, flip angle 11°, readout bandwidth 260 Hz/pixel). Each volume took 2.13 minutes to acquire. Application of zero-filling interpolation doubled the nominal in-plane resolution (0.25 × 0.25 mm^2^).

At the identical level, a high-resolution diffusion tensor imaging (DTI) dataset was acquired with a cardiac-gated reduced-FOV single-shot spin-echo echo planar imaging sequence with outer volume suppression^16^ to assess microstructural changes of the whole spinal cord. Four measurements of 6 b = 0 (T2-weighted) and 30 b = 500 s/mm^2^ volumes were acquired, resulting in 144 images per participant and a nominal acquisition time of 6.17 minutes. The following parameters were applied: repetition time of 350 milliseconds; echo time of 71 milliseconds; slice thickness of 5 mm (10% interslice gap); resolution of 0.76 × 0.76 mm^2^; FOV of 133 × 30 mm^2^; phase oversampling of 50%; 5/8 partial-Fourier imaging in the phase-encoding direction; cardiac trigger delay of 200 milliseconds; and minimal time between triggers of 1,800 milliseconds. After acquisition, zero-filling interpolation was used to double the in-plane resolution (0.38 × 0.38 mm^2^).

### Image processing

#### Lesion segmentation

With the use of the Jim 6.0 software (Xinapse Systems, Aldwincle, UK), the lesion was segmented on the midsagittal T2-weighted images, being visible as a high-signal-intensity area within the spinal cord, as previously described.^7^ The following parameters were quantified: midsagittal anterior-posterior lesion width (equal to the maximal anterior-posterior width of the lesion), midsagittal rostrocaudal lesion length (equal to the maximal caudocranial extent of the lesion), total midsagittal lesion area, and midsagittal thickness of midsagittal ventral and dorsal tissue bridges at the widest point of the lesion, which was summed up to the total amount of midsagittal tissue bridges.

#### Processing of high-resolution macrostructural data above the level of injury

We used serial longitudinal registration^17^ embedded within SPM12 to average the five 3-dimensional MEDIC volumes, accounting for intraparticipant motion. To further increase the signal-to-noise ratio, the average volume was resampled at a double slice thickness. We then used the Jim 6.0 software to measure cross-sectional spinal cord area (SCA) of 3 slices. After the midpoint of the spinal cord was marked manually in each slice, the SCA was calculated automatically with the semiautomatic 3-dimensional active-surface model.^18^ Gray matter area (GMA), dorsal column area (DCA), VHA (approximately lamina VI–IX), and DHA (approximately lamina I–V) were extracted manually.^6^ White matter area (WMA) was calculated by subtracting the GMA from the SCA. The mean interobserver reliability and intraobserver reliability for these measures were previously shown.^8,16^

#### Preprocessing and estimation of DTI data

All processing of the DTI data was carried out with a modified version of the MatLab-based ACID toolbox optimized for the spinal cord. First, we reduced the in-plane FOV to 24 × 24 mm^2^ to exclude much of the non–spinal cord tissue in each participant. Then, DTI volumes were slice-wise linearly registered with 3 *df* (translation in the frequency- and phase-encoding direction, scaling in the phase-encoding direction) to correct for intraparticipant motion and eddy-current artifacts.^19^ A diffusion tensor was fitted by use of a robust tensor fitting algorithm that accounts for outlier volumes due to motion and physiologic artifacts.^20^ The following DTI index maps were extracted: fractional anisotropy (FA) and mean, axial, and radial diffusivity (MD, AD, and RD).

These DTI index maps were then spatially normalized to a self-constructed mean diffusivity template residing in the spinal Montreal Neurological Institute space.^21^ To further refine the accuracy of the registration, a manual slice-by-slice registration (in-plane translation and scaling) was performed. Finally, all DTI index maps were smoothed with a full width at half-maximum gaussian kernel with 0.5 × 0.5 × 5 mm^3^.

### Statistical analysis

Statistical analysis of all macrostructural MRI, neurophysiologic, and clinical data was performed with Stata13 (StataCorp LP, College Station, TX). The mean age was not statistically different between healthy controls and patients (Mann-Whitney *U* test: *z* = −1.61, *p* = 0.10). All images were visually inspected for artifacts, and the analysis was conducted on 3 slices from each modality at the same level.

First, we assessed the morphometric differences in SCA, GMA, WMA, DCA, VHA, and DHA between patients and healthy controls by means of analysis of covariance, adjusted for age. For microstructural differences between patients and healthy controls, we used SPM12 for voxel-based analysis of the different DTI indexes (FA, MD, AD, RD) by means of analysis of covariance, adjusted for age. All statistical parametric maps were initially thresholded with a cluster-defining threshold of *p* < 0.01 (uncorrected) and clusters surpassing a cluster threshold of *p* < 0.05 (family-wise error corrected) are reported. Next, we used linear regression analysis to investigate the relationship between changes at the lesion site (midsagittal lesion area, length and width, and size of midsagittal tissue bridges) and remote cord macrostructural and microstructural changes. We then determined associations between macrostructural (SCA, GMA, WMA, DCA, VHA, and DHA) and microstructural (DTI indexes within lateral corticospinal tract, dorsal column, and spinal lemniscus) parameters and tract-specific clinical measures (motor, pinprick, and light-touch score, GRASSP, SCIM) using linear regression models, adjusted for age and lesion area. Finally, we investigated associations between macrostructural and microstructural MRI indexes and neurophysiologic outcome measures using linear regression models, adjusted for age and lesion area. Note that only patients with both recordable electrophysiologic potentials and available MRI data entered this regression analysis, resulting in a total number of 8 patients. For all microstructural associations, we extracted mean values of DTI indexes within anatomic regions of interest (lateral corticospinal tract, dorsal column, and spinal lemniscus [containing spinothalamic and spinoreticular tracts]) as embedded in the Spinal Cord Toolbox.^22^ The level of significance was set to *p* < 0.05.

## Results

### Radiologic, clinical, and neurophysiologic characteristics

**Figure 1:**
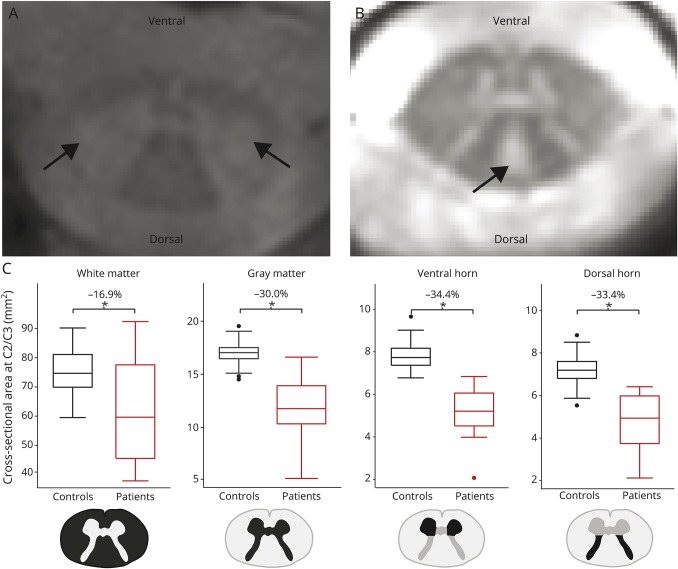
Macrostructural changes above the level of injury Hyperintense regions most likely indicating (A) retrograde degeneration in the corticospinal tract and (B) anterograde degeneration in the dorsal column. Arrows indicate the corresponding locations. (C) Differences between the cross-sectional white matter area, cross-sectional gray matter area, cross-sectional ventral horn area, and cross-sectional dorsal horn area in patients compared to healthy controls.

Patients were scanned 6.7 ± 7.8 years after injury. An area of hyperintense signal was visible on the T2-weighted sagittal images in 16 patients (figure 1, A and B); 13 patients showed hyperintensities in their dorsal column, covering on average 41.4 ± 21.0% of the whole dorsal column, and 2 patients showed hyperintensities in the dorsolateral funiculus (e.g., corticospinal tract). The radiologic level of injury (hyperintense T2-weighted signal) covered the vertebral level C3-5 in 1 patient, C3 in 1 patient, C4 in 2 patients, C4-5 in 1 patient, C5 in 2 patients, C6 in 2 patients, C6-7 in 4 patients, and C7 in 2 patients. Two patients showed no signal alteration within the cord. The average lesion area was 45.4 ± 66.6 mm^2^ with a lesion length of 11.3 ± 9.4 mm and a lesion width of 4.3 ± 3.5 mm. In 2 patients, the lesion occupied the full cord area, and no midsagittal tissue bridges could be identified. In the remaining 15 patients, the midsagittal tissue bridges had an average width of 2.9 ± 1.9 mm. No magnetic resonance abnormalities were identified in the control group.

Two patients were motor and sensory complete; 2 patients were motor complete and sensory incomplete; and the remaining 13 patients were motor and sensory incomplete. The motor score (maximum 100) was 68.1 ± 30.4; the light-touch score was (mean ± SD) 66.3 ± 32.7 (maximum 112); and the pinprick score (maximum 112) was 52.7 ± 35.0. Manual dexterity was impaired as assessed by the GRASSP score (149.8 ± 66.3 [maximum 232]), and functional independence was impaired as assessed by the SCIM score ([63.1 ± 31.3 [maximum. 100]). Eight patients were able to walk independently (20 of 20 points in the WISCI score); 2 patients were dependent on walking aids (5 of 20 and 9 of 20 points in the WISCI score); and 7 patients were not able to walk (0 of 20 points). All data are summarized in table 1.

**Table 1:**
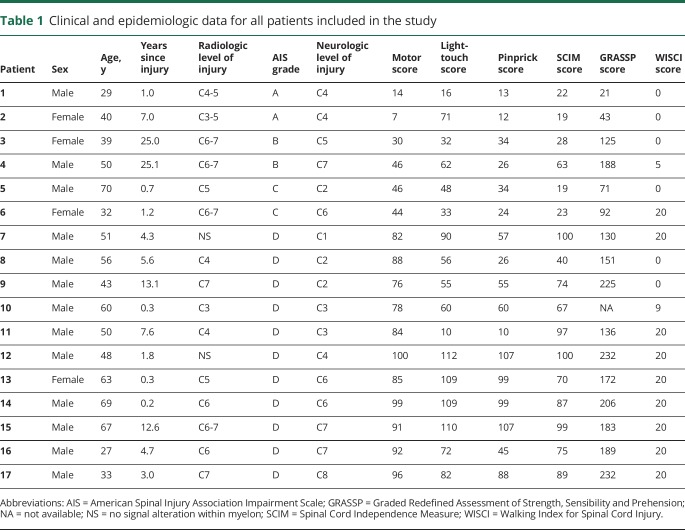
Clinical and epidemiologic data for all patients included in the study

All patients had neurophysiologic impairment of the spinothalamic tract, and a majority had impaired function of the dorsal column below the level of lesion as assessed by CHEPs and SSEPs, respectively. The mean ± SD perception/pain thresholds and the amplitudes and latencies of the recorded signals are shown in table 2.

**Table 2:**
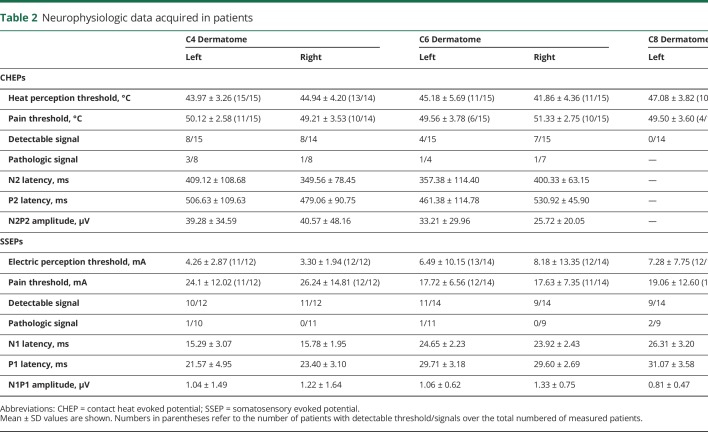
Neurophysiologic data acquired in patients

### Pathophysiologic changes in the cervical cord above the level of injury

Compared to healthy controls, patients showed a decreased SCA of 20.2% (*p* < 0.001, healthy controls 92.30 ± 8.49 mm^2^, patients 73.71 ± 20.04 mm^2^). In patients, WMA was decreased by 16.9% (*p* = 0.001, healthy controls 75.34 ± 8.06 mm^2^, patients 62.64 ± 18.22 mm^2^), and GMA was decreased by 30.0% (*p* < 0.001, healthy controls 16.96 ± 1.25 mm^2^, patients 11.93 ± 2.73 mm^2^). In the white matter, DCA was decreased by 21.4% (*p* < 0.001, healthy controls 23.73 ± 2.99 mm^2^, patients 18.65 ± 4.76 mm^2^). Within the gray matter, the bilateral VHA showed a 34.4% decrease in patients compared to healthy controls (left: *p* < 0.001, healthy controls 3.84 ± 0.29 mm^2^, patients 2.56 ± 0.62 mm^2^; right: *p* < 0.001, healthy controls 3.95 ± 0.40 mm^2^, patients 2.61 ± 0.58 mm^2^). In patients, the DHA was decreased bilaterally by 33.4% (left: *p* < 0.001, healthy controls 3.63 ± 0.41 mm^2^, patients 2.42 ± 0.66 mm^2^; right: *p* < 0.001, healthy controls 3.60 ± 0.51 mm^2^, patients 2.37 ± 0.66 mm^2^) (figure 1C), and smaller DHA was associated with smaller DCA (*p* < 0.001, *R*^2^ = 0.74, 95% confidence interval [CI] 2.21–4.28).

**Figure 2:**
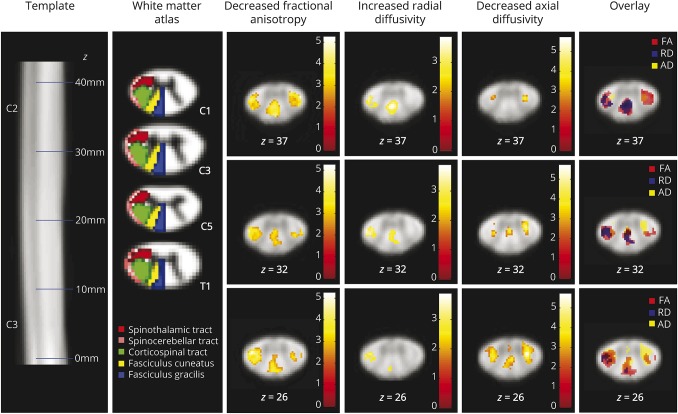
Microstructural changes above the level of injury Voxel-wise analysis of microstructural changes above the level of injury in patients compared to healthy controls. First row shows the spinal cord template; second row shows the white matter atlas. Note the spatial overlap of the different diffusion tensor imaging metrics showing that regions of decreased axonal diffusivity (AD; e.g., axonal degeneration) but unaltered radial diffusivity (RD; e.g., no demyelination) lie mostly adjacent to the gray matter, where unmyelinated propriospinal neurons are located. FA = fractional anisotropy. Reprinted from De Leener et al^22^ with permission from Elsevier.

Voxel-based analysis of the cervical cord revealed a 16.6% decrease in FA in the left dorsolateral funiculus (e.g., containing spinothalamic and lateral corticospinal tracts, *p* = 0.003; localization [x, y, z] −4.2, −18.5, 26; *z* score 4.42; cluster extent 154), 14.9% decrease in the right dorsolateral funiculus (*p* = 0.025; localization [x, y, z] 6, −18.5, 37; *z* score 4.34; cluster extent 85), and 17.0% decrease in the posterior funiculus (i.e., containing dorsal columns; *p* = 0.004; localization [x, y, z] 0.7, −22.3, 37; *z* score 3.80; cluster extent 145) in patients compared to healthy controls. AD was also decreased in patients compared to healthy controls in the same regions, namely by 12.8% in the left dorsolateral funiculus (*p* = 0.014; localization [x, y, z] −3.1, −19.2, 26; *z* score 3.72; cluster extent 58), 12.8% the right dorsolateral funiculus (*p* = 0.002; localization [x, y, z] 4.1, −18.8, 26; *z* score 4.70; cluster extent 94), and 9.9% in the posterior funiculus (*p* = 0.020; localization [x, y, z] 0.7, −19.2, 32; *z* score 3.69; cluster extent 52). RD increased by 31.8% in the dorsal column (*p* = 0.022; localization [x, y, z]: 0.3, −20.7, 37; *z* score 3.47; cluster extent 70) and by 34.0% in the left dorsolateral funiculus (*p* = 0.023; localization [x, y, z] −5, −19.2, 32; *z* score 3.22; cluster extent 69) in patients compared to healthy controls. MD was not significantly different between patients and healthy controls (figure 2).

### Relationship between lesion severity and remote tissue-specific neurodegeneration

**Figure 3:**
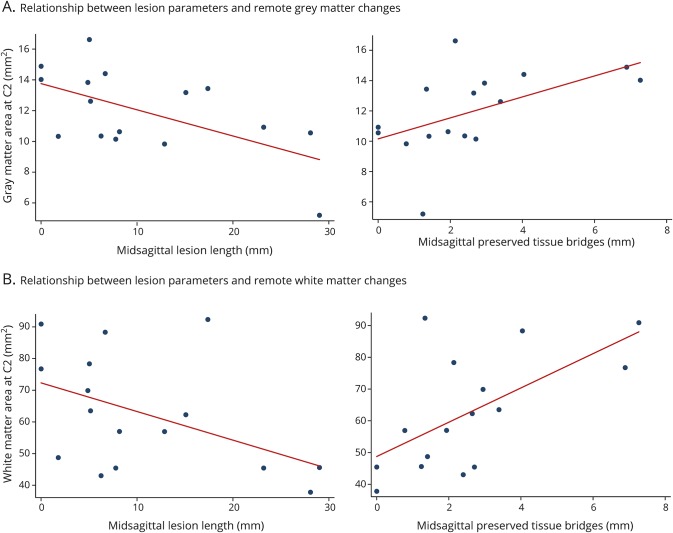
Relationship between lesion severity and neurodegeneration above the level of injury The magnitude of tissue damage at the lesion site is associated with the amount of neurodegeneration above the level of injury. Lesion length and midsagittal tissue bridges are associated with (A) remote gray matter and (B) white matter atrophy.

Greater lesion area and length were associated with greater SCA decrease above the level of injury (lesion area: *p* = 0.048, *R*^2^ = 0.25, 95% CI −3.64 to −0.23 1/mm^3^; lesion length: *p* = 0.006, *R*^2^ = 0.42, 95% CI −0.55 to −0.11 1/mm^2^) independently of age. The width of total midsagittal tissue bridges was associated with less SCA decrease (*p* = 0.007, *R*^2^ = 0.39, 95% CI 0.02–0.11 1/mm^2^). Greater lesion length was associated with smaller GMA (*p* = 0.012, *R*^2^ = 0.40, 95% CI −3.74 to −0.57 1/mm^2^), VHA (*p* = 0.039, *R*^2^ = 0.29, 95% CI −8.33 to −0.25 1/mm^2^), and DHA (*p* = 0.004, *R*^2^ = 0.49, 95% CI −8.36 to −2.03 1/mm^2^), while midsagittal tissue bridges were positively associated with GMA (*p* = 0.035, *R*^2^ = 0.28, 95% CI 0.33–0.781/mm^2^) and DHA (*p* = 0.011, *R*^2^ = 0.38, 95% CI 0.26–1.74 1/mm^2^) (figure 3A). Greater lesion length and preserved midsagittal tissue bridges were associated with WMA (lesion length: *p* = 0.014, *R*^2^ = 0.38, 95% CI −0.61 to −0.08 1/mm^2^; tissue bridges: *p* = 0.011, *R*^2^ = 0.38, 95% CI 0.02–0.12 1/mm^2^) above the level of injury (figure 3B).

The width of total midsagittal tissue bridges was associated with DCA above the level of lesion (*p* = 0.019, *R*^2^ = 0.29, 95% CI 0.25–2.39 1/mm^2^). Neither lesion size nor midsagittal tissue bridges were associated with microstructural changes above the level of lesion.

### Relationship between remote neurodegeneration and neurophysiologic outcome

**Figure 4:**
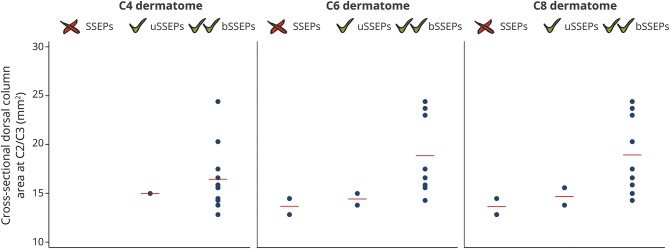
Relationship between neurodegeneration above the level of injury and electrophysiologic outcome Patients were grouped into 3 cohorts: without or with unilateral (u) or bilateral (b) recordable dermatomal somatosensory-evoked potentials (SSEPs) at the C4, C6, and C8 level. Patients with recordable dermatomal SSEPs showed a tendency toward larger dorsal column area above the level of injury.

The size of the cross-sectional area of the dorsal columns identified those patients with bilateral recordable SSEPs of the dermatomes C6 and C8 (figure 4). This relationship was not evident for the WMA and CHEPs. Higher AD values within the dorsal column were associated with shorter SSEP N1P1 latency at the C4 dermatome (*p* = 0.0024, *R*^2^ = 0.83, 95% CI: −0.00007 to −0.00001 10^−3^ × s^2^/mm^2^), corrected for age and lesion area. DTI metrics within the spinothalamic tracts were not associated with CHEPs recordings.

### Relationship between remote neurodegeneration and clinical outcome

**Figure 5:**
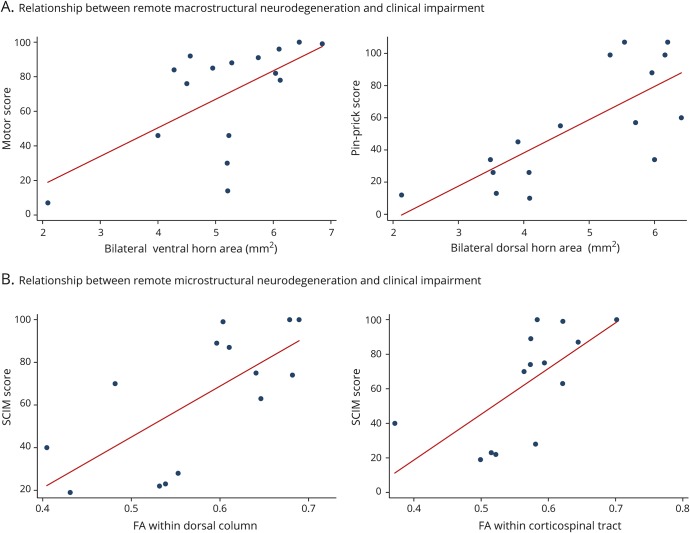
Relationship between neurodegeneration above the level of injury and clinical outcome Associations between (A) remote tract-specific macrostructural MRI parameters above the level of injury and clinical impairment and (B) remote tract-specific microstructural MRI indexes above the level of injury and clinical impairment. FA = fractional anisotropy; SCIM = Spinal Cord Independence Measure.

GMA was associated with motor score (*p* = 0.007, *R*^2^ = 0.72, 95% CI 1.77–9.26) and pinprick score (*p* = 0.003, *R*^2^ = 0.58, 95% CI 3.48–13.90); VHA area was associated with motor score (*p* = 0.001, *R*^2^ = 0.78, 95% CI 6.74–21.93); and DHA was associated with pinprick score (*p* = 0.004, *R*^2^ = 0.57, 95% CI 7.43–31.52) when corrected for lesion area and age (figure 5A).

To quantify tract-specific associations with appropriate clinical outcome, we used the extracted mean values of DTI indexes within the regions of interest (i.e., corticospinal tract, dorsal column, and spinothalamic tract). FA and RD within corticospinal tract and the dorsal columns were associated with SCIM score (corticospinal tract: FA: *p* = 0.002, *R*^2^ = 0.80, 95% CI 105.82–361.55; RD: *p* = 0.001, *R*^2^ = 0.83, 95% CI −169829.70 to −60547.48; dorsal columns: FA: *p* = 0.002, *R*^2^ = 0.80, 95% CI 106.07–341.42; RD: *p* = 0.003, *R*^2^ = 0.79, 95% CI −216944.1 to −60854.36) independently of lesion extent and age (figure 5B). AD within the dorsal columns was associated with GRASSP score independently of lesion extent and age (*p* = 0.30, *R*^2^ = 0.60, 95% CI 40298.4–646999.7).

## Discussion

This study shows the in vivo structure-function relationship between the extent of tissue-specific cord pathology and neurophysiologic and clinical impairment after traumatic SCI. Crucially, we show that the magnitude of tissue damage at the lesion epicenter is associated with the extent of neurodegeneration above the level of lesion, which, in turn, is associated with clinically relevant impairment and neurophysiologic abnormalities. These findings allow us to investigate the extent of tissue-specific neurodegeneration above the level of injury, its relationship to neuronal tissue loss at the site of the lesion, and its effect on neurophysiologic and clinical outcome.

Tissue damage at the epicenter of a traumatic SCI results both from the direct effect of the traumatic insult and from damage to the vascular architecture and the ensuing ischemic effects on the neuronal and glial cell populations within the acute phase of injury.^23^ Remote from the epicenter of the lesion, secondary neurodegeneration within white^24,25^ and gray matter^26^ follows with a time lag and is driven by a multiphasic response to cellular inflammation.^27^ While the extent of secondary remote atrophy has been quantified in vivo after injury,^2,28–30^ we provide evidence that changes within both gray and white matter contribute to cord atrophy above the level of injury. This is in agreement with spinal gray matter degeneration distant to the initial site of damage in patients with multiple sclerosis^31^ and experimental SCI.^32^ Although the relative decrease is larger within ventral and dorsal horns (i.e., gray matter), the absolute magnitude of change is larger within white matter, contributing more to the overall loss of SCA by 20.2%.

We uncovered an in vivo relationship between neuronal tissue loss (i.e., lesion severity) and remote tissue-specific cord pathology above the level of injury. Moreover, we show an interdependence of remote white and gray matter atrophy (i.e., DHA and DCA). Neurodegenerative changes within gray matter above the level of injury are not likely to be specific for any single pathologic process but rather are likely to represent a combination of different pathologic mechanisms taking place after SCI. Possible mechanisms involve transsynaptic/transneuronal degeneration affecting the propriospinal systems^33,34^ and motor neurons located in the proximity of the spinal injury.^35^ Next to direct effects of neurodegenerative processes, a reduction in muscle activity of the upper extremity could lead to a reduction of neuronal activity above the level of injury, which may translate into shrinkage of the neuron soma size.

Furthermore, demyelination of corticospinal projections to the dorsal horns,^36^ the expression of neurotrophic factors from nonneuronal cells around neighboring degenerating axons,^36^ growth factor dysregulation,^37^ and vascular remodeling^38^ could contribute to gray matter pathology. As white matter damage is known to induce microglial activation altering glutamate signaling, this process is thought to be responsible for the dying back of axons and their parternal neurons,^39^ which might be a shared underlying disease mechanism. Thus, next to anterograde and retrograde degeneration of white matter tracts^30,40,41^ the neuronal circuits within the spinal cord far above the level of injury undergo a temporary structured neurodegeneration.^25^

Within the microstructure of the atrophied white matter, we found indications of both axonal degeneration and demyelination,^42^ represented by decreased FA and AD and increased RD in the dorsolateral funiculus (e.g., containing the lateral corticospinal and spinothalamic tracts) and posterior funiculus (e.g., containing the dorsal columns). Within the corticospinal tract and the dorsal column, leg function is represented most laterally, whereas arm function is located either medially (i.e., corticospinal tract) or centrally (i.e., dorsal column). Our observed changes cover the entire lateral corticospinal tract and the dorsal columns, indicating neurodegenerative processes affecting axons that convey information relating to leg and arm function. Spatially overlaying the different DTI metrics changes revealed that regions showing decreased AD (e.g., axonal degeneration) but unaltered RD (e.g., no demyelination) lie mostly adjacent to the gray matter border. This region contains the fasciculi proprii and contains mostly short, mainly unmyelinated propriospinal neurons.^43^ This underlies our hypothesis that SCI might lead to degeneration affecting interneurons within the spinal cord.

Our findings complement previous studies in patients with SCI ^20,44–46^ in that they now locate these changes to gray and white matter rather than being nonspecific in terms of location and tissue. Thus, our in vivo cord MRI measurements demonstrate a combination of structural and functional processes occurring over several segments above the level of injury affecting both gray and white matter that is driven by lesion severity.

We show that tract-specific microstructural and macrostructural changes are associated with prolonged conduction of appropriate electrophysiologic recordings. This association suggests a structure-function relationship because the amount of neurodegeneration was directly associated with impairment of neurophysiologic information flow. Because the DTI signal is sensitive to altered diffusion properties occurring as a response to CNS damage (i.e., demyelination/degeneration) and because neuronal excitability is affected by morphometry of the axon and its myelin, it seems plausible that neurophysiologic changes might be reflected in remote macrostructural and microstructural changes above the level of injury. Our findings complement previous findings showing that the topography and the excitability of corticomotor projections were associated with cervical cord atrophy.^2,47^

Current assessments in patients with SCI lack sensitivity to minimal changes in motor and sensory function^48^ in that they cannot detect subtle changes due to remyelination and axonal regeneration. Neuroimaging biomarkers have the potential to track these subtle abnormalities because they are sensitive to microstructural changes.^1^ In this study, the magnitude of both remote macrostructural and microstructural changes within gray and white matter was significantly associated with clinical impairment, independently of lesion extent. In particular, the extent of remote ventral horn atrophy was associated with motor impairment, whereas dorsal horn atrophy was associated with sensory disturbance. Microstructural tract-specific changes above the level of injury were related to measures of functional independence (i.e., SCIM) and strength, sensibility, and prehension of the upper limbs (i.e., GRASSP). This suggests that high-resolution MRI sequences applied above the level of injury provide superior information on the patient's clinical status compared to standard clinical sequences at the lesion site. In addition, the latter findings are striking in that they suggest that remote neurodegeneration within gray matter above the level of injury contributes, in addition to white matter pathology, to motor and sensory impairment. This multilevel interaction supports the view that SCI leads to a cascade of neurodegenerative changes affecting the entire spinal cord and brain.^49^ Characterizing these secondary neurodegenerative events has the potential to provide insights into new therapeutic interventions, in addition to providing opportunities for monitoring treatment effects in trials conducted in patients with acute and chronic SCI.

This study had several limitations. Although our cohorts did not show a significant age difference, the mean age was on average higher in the patient group, which could potentially affect the analysis. We therefore adjusted for age as a potential confounder of no interest in all analyses. Furthermore, unbiased voxel-based morphometry of DTI indexes in the spinal cord has just started emerging,^3^ and the automated postprocessing methods for spatial normalization of the spinal cord into common space are in their infancy. To increase the reliability of our analysis, we therefore manually corrected the spatial normalization to the template.

This study shows that the magnitude of dorsal and ventral horn and white matter structural changes above the level of injury is associated with appropriate clinical and neurophysiologic impairment and is driven by lesion severity. These findings suggest a combination of different pathologic processes affecting both gray and white matter several segments above the level of injury that are clinically eloquent. Therefore, these neuroimaging biomarkers might serve as promising surrogate markers for future clinical trials supplementing (or complementing) clinical outcome measures.

## Glossary

AD: axonal diffusivity
CHEP: contact heat evoked potential
CI: confidence interval
DCA: dorsal column area
DHA: dorsal horn area
DTI: diffusion tensor imaging
FA: fractional anisotropy
FOV: field of view
GMA: gray matter area
GRASSP: Graded Redefined Assessment of Strength, Sensibility and Prehension
MD: mean diffusivity
RD: radial diffusivity
SCA: spinal cord area
SCI: spinal cord injury
SCIM: Spinal Cord Independence Measure
SSEP: somatosensory evoked potential
VHA: ventral horn area
WISCI: Walking Index for Spinal Cord Injury
WMA: white matter area
